# Degradation of 4-Tert-Butylphenol in Water Using Mono-Doped (M1: Mo, W) and Co-Doped (M2-M1: Cu, Co, Zn) Titania Catalysts

**DOI:** 10.3390/nano12142326

**Published:** 2022-07-06

**Authors:** Saule Mergenbayeva, Alisher Kumarov, Timur Sh. Atabaev, Evroula Hapeshi, John Vakros, Dionissios Mantzavinos, Stavros G. Poulopoulos

**Affiliations:** 1 Department of Chemical and Materials Engineering, School of Engineering and Digital Sciences, Nazarbayev University, 53 Kabanbay Batyr Ave., Nur-Sultan 010000, Kazakhstan; saule.mergenbayeva@nu.edu.kz (S.M.); alisher.kumarov@nu.edu.kz (A.K.); 2 Department of Chemistry, School of Sciences and Humanities, Nazarbayev University, 53 Kabanbay Batyr Ave., Nur-Sultan 010000, Kazakhstan; timur.atabaev@nu.edu.kz; 3 Department of Life and Health Sciences, School of Sciences and Engineering, University of Nicosia, 2417 Nicosia, Cyprus; hapeshis.e@unic.ac.cy; 4 Department of Chemical Engineering, University of Patras, Caratheodory 1, University Campus, GR-26504 Patras, Greece; vakros@chemistry.upatras.gr (J.V.); mantzavinos@chemeng.upatras.gr (D.M.)

**Keywords:** 4-tert-butylphenol, titanium dioxide, transition metals, photocatalytic degradation

## Abstract

Mono-doped (Mo-TiO_2_ and W-TiO_2_) and co-doped TiO_2_ (Co-Mo-TiO_2_, Co-W-TiO_2_, Cu-Mo-TiO_2_, Cu-W-TiO_2_, Zn-Mo-TiO_2_, and Zn-W-TiO_2_) catalysts were synthesized by simple impregnation methods and tested for the photocatalytic degradation of 4-tert-butylphenol in water under UV (365 nm) light irradiation. The catalysts were characterized with various analytical methods. X-ray diffraction (XRD), Raman, Diffuse reflectance (DR) spectroscopies, Scanning electron microscopy (SEM), Transmission electron microscopy (TEM), and Energy dispersive spectroscopy (EDS) were applied to investigate the structure, optical properties, morphology, and elemental composition of the prepared catalysts. The XRD patterns revealed the presence of peaks corresponding to the WO_3_ in W-TiO_2_, Co-W-TiO_2_, Cu-W-TiO_2_, and Zn-W-TiO_2_. The co-doping of Cu and Mo to the TiO_2_ lattice was evidenced by the shift of XRD planes towards higher 2θ values, confirming the lattice distortion. Elemental mapping images confirmed the successful impregnation and uniform distribution of metal particles on the TiO_2_ surface. Compared to undoped TiO_2_, Mo-TiO_2_ and W-TiO_2_ exhibited a lower energy gap. Further incorporation of Mo-TiO_2_ with Co or Cu introduced slight changes in energy gap and light absorption characteristics, particularly visible light absorption. In addition, photoluminescence (PL) showed that Cu-Mo-TiO_2_ has a weaker PL intensity than undoped TiO_2_. Thus, Cu-Mo-TiO_2_ showed better catalytic activity than pure TiO_2_, achieving complete degradation of 4-tert-butylphenol under UV light irradiation after 60 min. The application of Cu-Mo-TiO_2_ under solar light conditions was also tested, and 70% of 4-tert-butylphenol degradation was achieved within 150 min.

## 1. Introduction

Industrialization on a large scale, along with urbanization and population growth, results in the development of vast volumes of wastewater with different pollutants (inorganic and organic). Numerous organic pollutants found in wastewater are hazardous and may pose a threat to the aquatic environment and living beings [1]. These pollutants include endocrine disrupting chemicals (EDC), pharmaceuticals, and personal care products (PPCPs) [2]. The presence of EDCs in water sources has become one of the major environmental issues [3]. Even at a low exposure level, they may cause the disruption of endocrine and reproductive systems [4]. 4-tert-Butylphenol (4-t-BP) is a synthetic EDC that has been widely utilized in the manufacture of polycarbonate, phenolic, and epoxy resins and, thus, is commonly detected in seas, rivers, sediments, and landfill leachate [5,6,7]. As a typical EDC, 4-t-BP was found to have poor biological degradability and high estrogenic activity [8,9]. Due to the persistence [10,11] and adverse effects of 4-t-BP on aquatic life [12] and living creatures, economically viable and sustainable technology is highly needed to eliminate 4-t-BP from water.

Several approaches have been investigated for the removal of 4-t-BP, including biological processes and advanced oxidation processes (AOPs) [11,13,14]. In contrast with the inefficiency of and relatively long time required by biological degradation, AOPs have received a lot of interest for their ability to remove such persistent pollutants by turning them into carbon dioxide and water [15,16,17]. AOPs are based on the generation of highly reactive radicals, such as hydroxyl radicals (•OH), that can easily react with organic compounds [18,19,20]. Heterogeneous photocatalysis is an AOP that has been successfully employed to remove different organic pollutants. The process is considered to be promising mainly due to its low cost and mild operating conditions, namely, ambient pressure and room temperature [21,22].

TiO_2_-based photocatalysts continue to be one of the most investigated materials, owing to their great photocatalytic activity, chemical stability, and availability [23,24]. TiO_2_ may be found in three different crystallographic forms, which are anatase, rutile, and brookite [25,26]. TiO_2_ in the P25 form is a mixture of anatase and rutile and is one of the most powerful photocatalytic materials. However, the large energy band gap of TiO_2_ (about 3.0–3.2 eV) prohibits its application under visible light. To extend its light absorption property to the visible light region, mono-doping and co-doping of TiO_2_ with various cationic and anionic impurities can substantially reduce the band gap energy and thereby improve photocatalytic efficiency in the visible light region [27,28].

The incorporation of transition metals with higher oxidation states, such as Mo^6+^ and W^6+^, into the TiO_2_ lattice has shown great promise due to the broadening of the spectral response and the ability to carry out visible light photocatalysis [29,30,31]. Both Mo^6+^ and W^6+^ have similar ionic radii to Ti^4+^; therefore, they are easy to introduce into the TiO_2_ lattice [32]. Additionally, the presence of Mo^6+^ has been proved to be beneficial for the formation of Ti^3+^ defect sites and suppressing charge carrier recombination, thanks to the redox potential of Mo^6+^/Mo^5+^ (vs. NHE), which is 0.4 V [33]. On the other hand, modification of TiO_2_ with W^6+^ may increase the surface acidity of the catalyst, leading to the absorption of more hydroxyl groups and pollutant molecules. Avilés-García et al. investigated the effect of Mo- and W- dopants on the performance of TiO_2_ [34]. The results demonstrated Mo-TiO_2_ and W-TiO_2_ to have better activity for 4-chlorophenol degradation than TiO_2_, attributed to the high surface area and enhanced charge separation [35].

In addition to Mo and W, modifying TiO_2_ with transition metals such as Cu, Co, and Zn could enhance the photocatalytic activity for the degradation of different organic pollutants in the UV–visible region by changing the physicochemical properties of TiO_2_ [36,37,38].

In this work, mono-doped (Mo-TiO_2_ and W-TiO_2_) and co-doped (Co-Mo-TiO_2_, Zn-Mo-TiO_2_, Co-W-TiO_2_, Cu-Mo-TiO_2_, Cu-W-TiO_2_, and Zn-W-TiO_2_) catalysts were synthesized through simple impregnation methods. The as-prepared samples were characterized by means of SEM, TEM, XRD, Raman, and UV–VIS DR spectroscopies to study their morphology, textural properties, crystal structure, and optical properties. Notably, this is the first time that ternary systems of TiO_2_ have been synthesized using two transition metals. Their photocatalytic activity was analyzed using 4-t-BP degradation under near-visible light (365 nm) and solar light irradiation.

## 2. Materials and Methods

### 2.1. Materials

4-t-BP (99%), applied as a target pollutant; titanium (IV) oxide (TiO_2_-P25, nanopowder, 21 nm primary particle size, ≥99.5%), used as the base photocatalyst; ammonium metatungstate ((NH_4_)_6_H_2_W_12_O_40_ xH_2_O analytical grade, CAS number 12333-11-8); ammonium heptamolybdate ((NH_4_)_6_Mo_72_O_24_ 4H_2_O analytical grade, CAS number 12054-85-2), cobalt nitrate (Co(NO_3_)_2_·6 H_2_O analytical grade, CAS number 10026-22-9), copper nitrate (Cu(NO_3_)_2_·3 H_2_O analytical grade, CAS number 10031-43-3), and zinc nitrate (Zn(NO_3_)_2_·6 H_2_O analytical grade, CAS number 10196-18-6), used for the deposition of the dopant metal; and NH_4_OH solution (28–30% ACS reagent, CAS Number 1336-21-6) were purchased from Sigma Aldrich (Saint Louis, MO, USA). The ultrapure water used in all experiments was obtained by means of a Direct-Q 3UV (Millipore, Darmstadt, Germany) water purification system. All chemical reagents were used without further purification.

### 2.2. Preparation of Photocatalysts

In this work, two base photocatalysts, namely, Mo/TiO_2_ and W/TiO_2_, were prepared using commercial TiO_2_ (Degussa P25, Saint Louis, MO, USA) as a support with the wet impregnation method, and the active species were W(VI) or Mo(VI) oxoanions using ammonium metatungstate and ammonium heptamolybdate, respectively. A proper amount of TiO_2_ (3.0 g) was suspended in the solution containing the required amount of Mo or W oxo species (2.5 × 10^–4^ moles Mo or W) for coverage with Mo or W of 1 at/nm^2^. The pH of the suspension was around 5 for the Mo solution, while, for the W solution, it was raised to 10 using the 28% NH_4_OH solution in order to depolymerize the polytungstate species and increase solubility. The suspension was place in a rotary evaporator and left under rotation for 90 min at 45 °C in order to maximize the amount of adsorption. Afterwards, a vacuum was applied and the water evaporated, followed by drying at 105 °C for 2 h and calcination at 400 °C for 5 h. The two base catalysts were then used to prepare ternary systems by dry impregnation. The third cation deposited was either Co(II), Cu(II), or Zn(II), using the corresponding nitrate salts, with the surface concentration of the M(II) ion set to 0.5 at/nm^2^ (4.15 × 10^–5^ moles M(II) ions for 1 g of M1/TiO_2_). After impregnation, the samples were dried and calcined under the same conditions as for the base catalysts.

### 2.3. Characterization

The phase composition of all of the prepared catalysts was evaluated by X-ray diffraction (XRD, Rigaku SmartLab automated multifunctional X-ray Diffractometer, Tokyo, Japan) using Cu Kα radiation in the scanning range of 10–80°. The Scherrer equation (1) [39] was used to determine the average size of the TiO_2_ nanoparticles:

(1) $$D=\frac{0.9\lambda }{\beta \cos\theta }$$

where D is the crystallite size of the catalyst, λ is the X-ray wavelength (1.54060 Å), β is the full width at half maximum of the diffraction peak, and θ is the diffraction angle.

Raman spectra were recorded using a Raman spectrometer (Horiba, LabRam HR evolution, Kyoto, Japan) with a 532 nm laser excitation. A transmission electron microscope (TEM, JEM-2100 from Jeol Ltd., Japan) and a scanning electron microscope (SEM, Carl Zeiss Auriga Cross Beam 540) equipped with an energy-dispersive spectroscopy (EDS) system were applied to perform surface morphology measurements and to analyze the elemental composition of the catalysts. The optical properties of the mono- and co-doped TiO_2_ nanoparticles were investigated by means of diffuse reflectance spectroscopy (DRS, Varian Cary 3, Palo Alto, CA, USA). The recombination behaviors of charge carriers for Cu-Mo-TiO_2_ were obtained via photoluminescence (PL) emission analysis performed on a fluorescence spectrophotometer (F-7000, Hitachi, Tokyo, Japan). The specific surface area (SSA) of the catalysts was determined from N_2_ adsorption isotherms in liquid N_2_ temperature in a Tristar 3000 porosimeter (Micromeritics, Norcross, GA 30093-2901, USA) with the BET method.

### 2.4. Experimental Procedure

The photodegradation of 4-t-BP was carried out using a photochemical reactor operated in batch mode (Lanphan industry, Zhengzhou City, Henan Province, China) under UV irradiation (365 nm). In a typical experiment, 10 mg of photocatalyst was added to 100 mL of 15 ppm 4-t-BP solution. Prior to irradiation, the solution was stirred for 90 min in the dark to achieve an adsorption–desorption equilibrium between catalyst and pollutant. At a regular time interval (every 30 min), the sample was taken out and filtered through a 0.22 µm Millex syringe filter to remove the photocatalyst for further analysis.

The same procedure was applied to test the photocatalytic activity of Cu-Mo-TiO_2_ under simulated solar light irradiation (LCS-100 solar simulator, Oriel, Newport, Darmstadt, Germany) within 150 min.

The concentration of 4-t-BP was measured by a high-performance liquid chromatography instrument (HPLC, Agilent 1290 Infinity II, Santa Clara, CA, USA) equipped with a SB-C8 column (2.1 mm × 100 mm, 1.8 µm). The mobile phase composition was methanol and ultrapure water (50:50, *v*/*v*), which were mixed to compose the mobile phase.

## 3. Results and Discussion

### 3.1. Characterization of Photocatalysts

The SSA of the pure TiO_2_ (P25) was measured to be equal to 54 m^2^g^−1^. The Mo-TiO_2_ catalysts maintained the SSA value (53 m^2^g^−1^) after the deposition of Mo species, while a second impregnation with either Co, Zn, or Cu resulted in an almost unchanged SSA (52 m^2^g^−1^) for all of the ternary Mo-TiO_2_ systems. This was expected, since the loading of the second metal ion is low, while the deposition of Mo species occurs mainly with adsorption or interfacial deposition [40].

On the other hand, the deposition of W oxo species decreased the SSA value to 47 m^2^g^−1^. The decrease in SSA value can be due to the lower contribution of adsorption in the deposition of W species in contrast with the Mo deposition. This is caused by the higher solution pH, which does not favor adsorption [41]. The deposition of the second metal ion had no influence on the SSA value (45 m^2^g^−1^).

**Figure 1:**
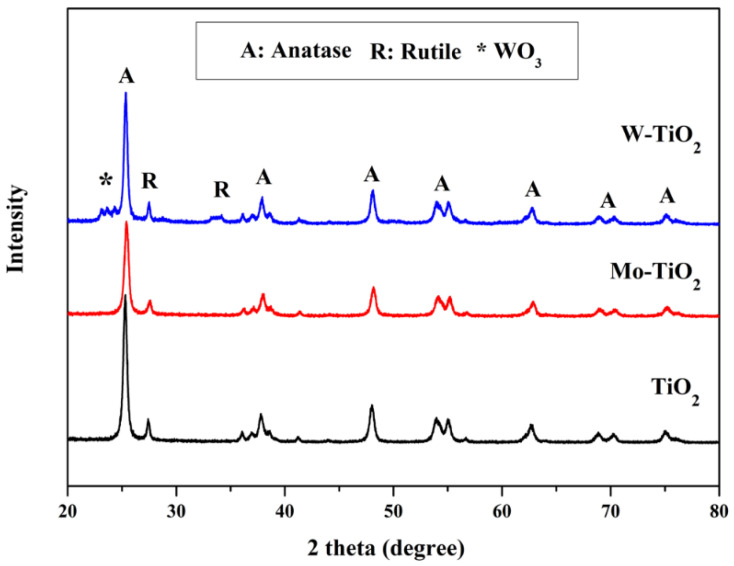
XRD patterns of Mo-TiO_2_ and W-TiO_2_.

**Figure 2:**
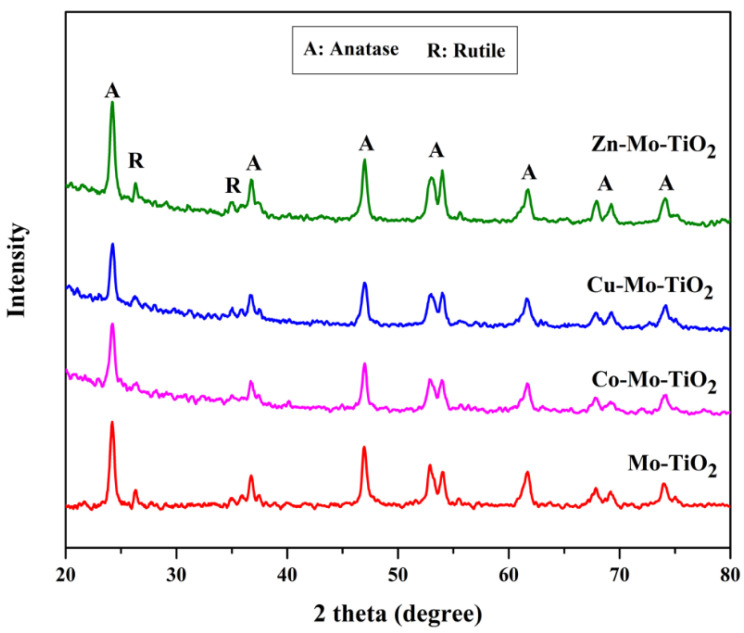
XRD patterns of Mo-TiO_2_, Co-Mo-TiO_2_, Cu-Mo-TiO_2_, and Zn-Mo-TiO_2_.

**Figure 3:**
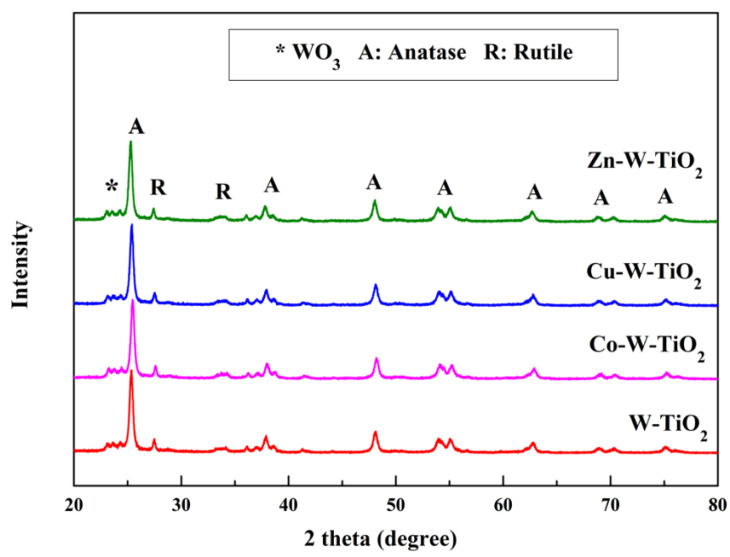
XRD patterns of W-TiO_2_, Co-W-TiO_2_, Cu-W-TiO_2_, and Zn-W-TiO_2_.

XRD analysis was performed to investigate the crystal structures of the mono- and co-doped TiO_2_ catalysts, and the results are shown in Figure 1, Figure 2 and Figure 3. The XRD patterns of all prepared catalysts were similar to that of pure TiO_2_, and a series of diffraction peaks corresponding to the anatase phase of TiO_2_ (ICDD Card No. 01-070-8501) can be recognized with the planes of (101), (004), (200), (105), (211), (204), (116), (220), and (215) at a degree of 2θ = 24.2°, 36.72°, 46.98°, 52.77°, 53.91°, 61.7°, 67.6°, and 73.92°, respectively. On the other hand, the peaks attributed to the rutile phase of TiO_2_ (ICDD Card No. 01-087-0920) were detected at around 2θ = 27.1° and 62°. Since the ionic radii of doped transition metals (Mo^6+^, W^6+^, Cu^2+^, Co^2+^, and Zn^2+^) are close to that of Ti^4+^ [42,43,44], minimum changes occurred in the original structure of TiO_2_.

The structure of the catalyst did not change drastically after the deposition of metals. No typical peaks were detected, which verified the impregnation of Mo-dopant into TiO_2_ (Figure 1) as well as the subsequent introduction of Cu, Co, and Zn particles into Mo-TiO_2_ (Figure 2). This finding could be attributed to the high dispersion of metal particles on the surface of TiO_2_ [45]. Interestingly, a slight shift of the intense TiO_2_ (101) peak towards a higher angle (from 24.2° to 24.6°) was observed only in Mo-Cu-TiO_2_, suggesting the existence of some disorders in the anatase crystal lattice [46,47].

Unlike Mo-doping, the introduction of W– into TiO_2_ formed new peaks at 2θ = 22.65° and 32.75° assigned to the WO_3_ phase (Figure 3). This is in accordance with the low decrease in SSA value for the W-TiO_2_ catalyst. The deposition of the second metal ion did not significantly alter the XRD pattern.

The average crystallite size of all prepared catalysts was calculated using Scherrer’s equation, and the results are listed in Table 1. The values confirmed the well-dispersed Mo phase and the existence of the nanoparticles on the prepared catalysts.

**Figure 4:**
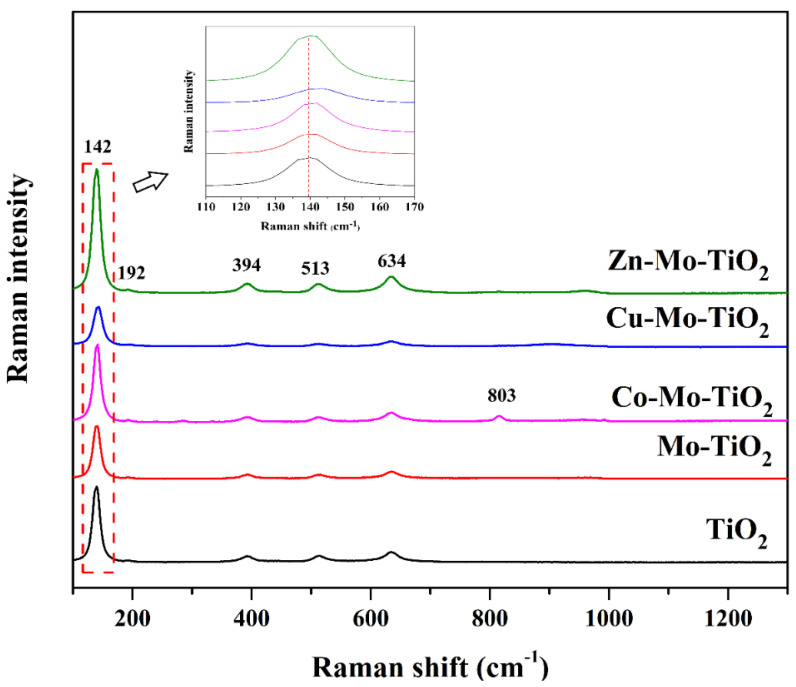
Raman spectra of TiO_2_, Mo− TiO_2_, Co− Mo−TiO_2_, Cu− Mo−TiO_2_, and Zn− Mo−TiO_2_.

**Figure 5:**
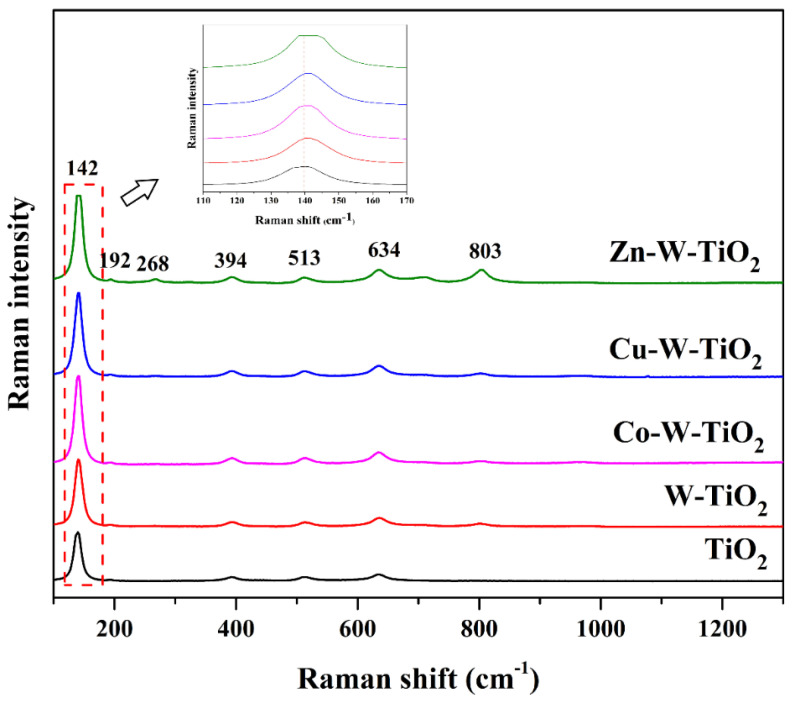
Raman spectra of TiO_2_, W−TiO_2_, Co−W−TiO_2_, Cu−W−TiO_2_, and Zn−W−TiO_2_.

Raman spectroscopy was used to obtain more information about the mono- and co-doped TiO_2_ nanoparticles, and the spectra in the range of 100–800 cm^−1^ are depicted in Figure 4 and Figure 5. Accordingly, the peaks located at 142 cm^−1^, 192 cm^−1^, 394 cm^−1^, 513 cm^−1^, and 634 cm^−1^ matched well with the anatase phase,_,_ while 268 cm^−1^ and 803 cm^−1^ confirm the presence of rutile phase. Any peaks corresponding to the doped transition metals could not be detected, although the shift of the main peak at 142 cm^−1^ towards a greater wavelength was observed for Cu-Mo-TiO_2_ and W-doped catalysts (W-TiO_2_, Cu-W-TiO_2_, Co-W-TiO_2_, and Zn-W-TiO_2_). The results of Raman spectroscopy related to the alternation in structure are in good agreement with experimental X-ray findings [48].

**Figure 6:**
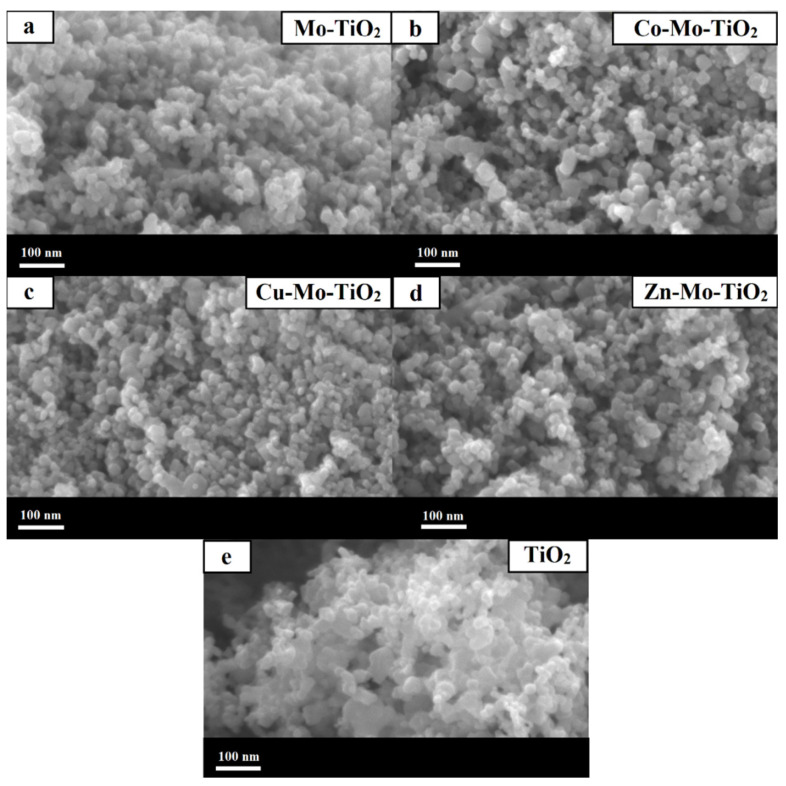
SEM images of (**a**) Mo-TiO_2_, (**b**) Co-Mo-TiO_2_, (**c**) Cu-Mo-TiO_2_, (**d**) Zn-Mo-TiO_2_, and (**e**) TiO_2_.

**Figure 7:**
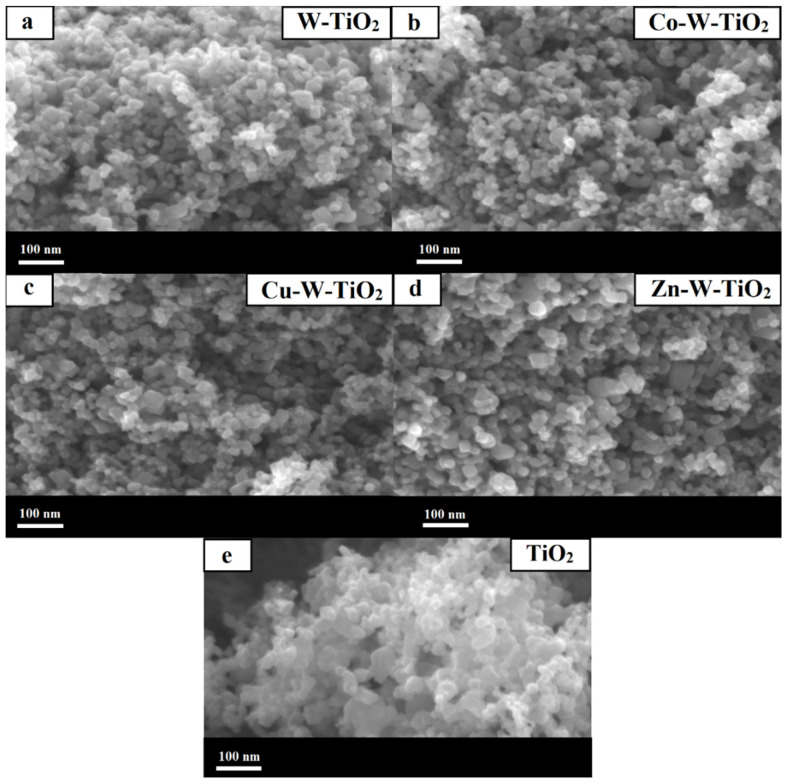
SEM images of (**a**) W-TiO_2_, (**b**) Co-W-TiO_2_, (**c**) Cu-W-TiO_2_, (**d**) Zn-W-TiO_2_, and (**e**) TiO_2_.

**Figure 8:**
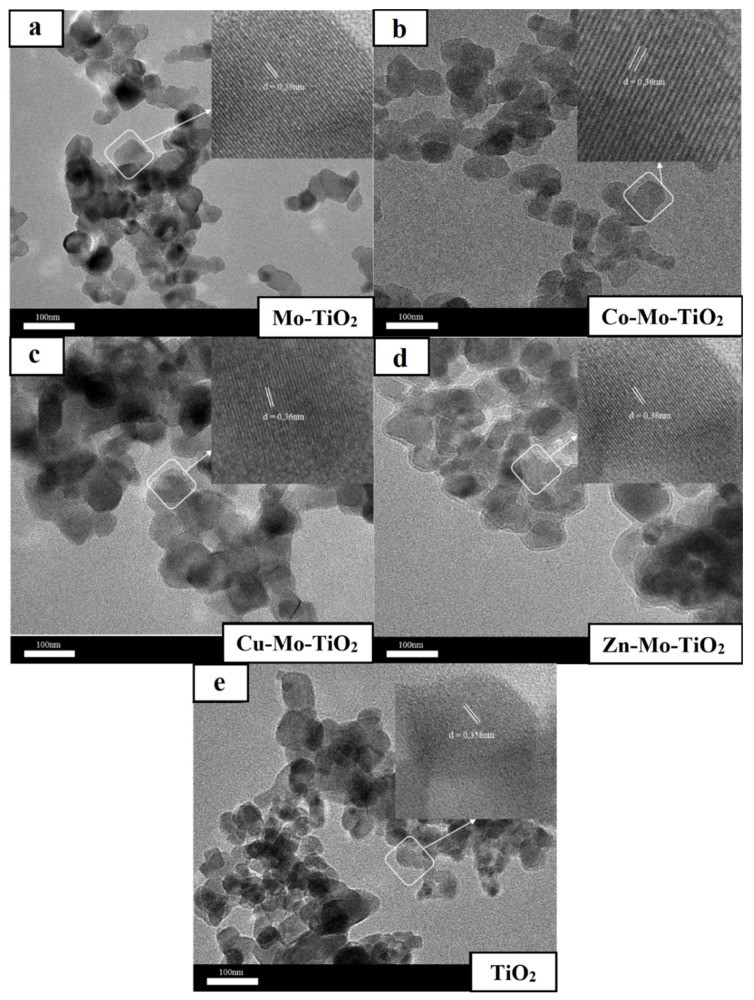
TEM analysis of (**a**) Mo-TiO_2_, (**b**) Co-Mo-TiO_2_, (**c**) Cu-Mo-TiO_2_, (**d**) Zn-Mo-TiO_2_, and (**e**) TiO_2_.

**Figure 9:**
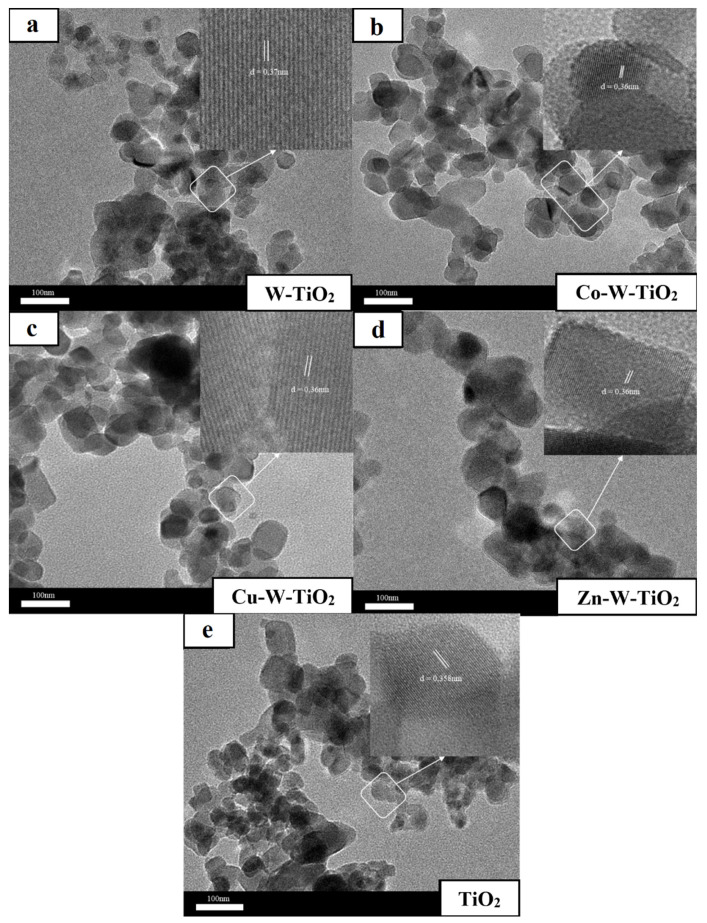
TEM analysis of (**a**) W-TiO_2_, (**b**) Co-W-TiO_2_, (**c**) Cu-W-TiO_2_, (**d**) Zn-W-TiO_2_, and (**e**) TiO_2_.

The surface morphology of the obtained catalysts was studied by both SEM and TEM analysis, depicted in Figure 6, Figure 7, Figure 8 and Figure 9. As can be seen from SEM images (Figure 6 and Figure 7), all prepared catalysts were found to be relatively spherical in shape, with particle sizes between 23 nm and 35 nm, like pure TiO_2_. These observations confirm the fact that the introduction of metals did not significantly affect the morphology of TiO_2_.

Notably, close-up TEM images (Figure 8 and Figure 9) reveal lattice spacing values of 0.37–0.41 nm that correspond to the [101] plane of TiO_2_ anatase. Overall, the results obtained from SEM and TEM characterizations (average particle size, crystal structure) are in good agreement with XRD and Raman findings.

**Figure 10:**
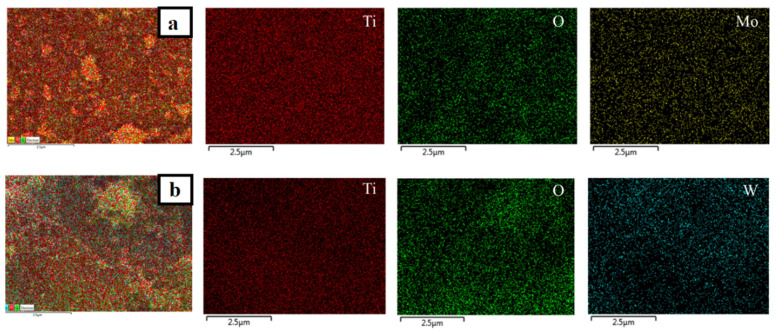
EDS mapping images of (**a**) Mo-TiO_2_ and (**b**) W-TiO_2_.

**Figure 11:**
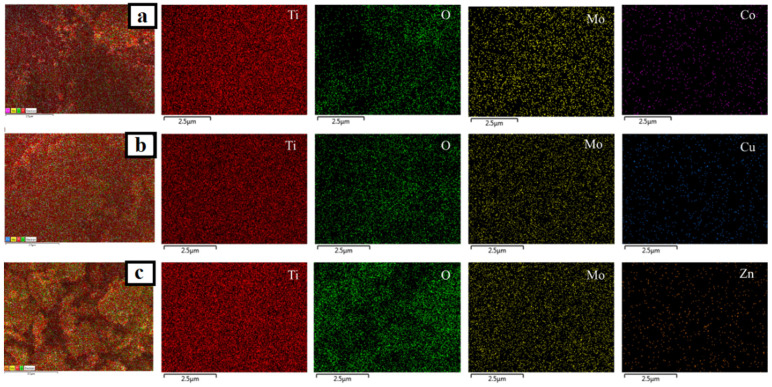
EDS mapping images of (**a**) Mo-Cu-TiO_2_, (**b**) Mo-Co-TiO_2_, and (**c**) Mo-Zn-TiO_2_.

**Figure 12:**
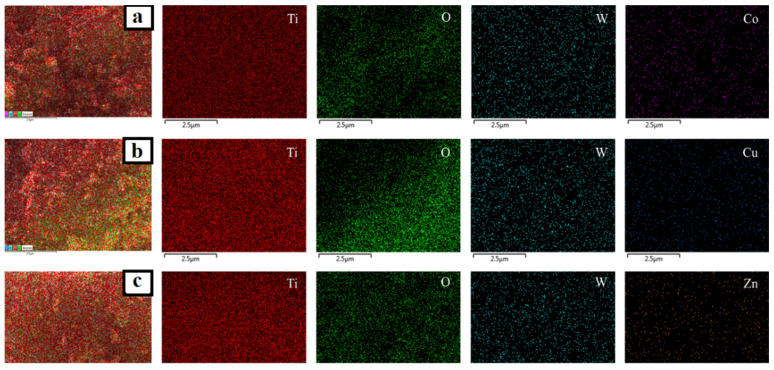
EDS mapping images of (**a**) W-Cu-TiO_2_, (**b**) W-Co-TiO_2_, and (**c**) W-Zn-TiO_2_.

In addition, EDS analysis was employed to investigate the elemental composition of the prepared catalysts. Although the doped metals were not visible as separate particles in TEM micrographs, EDS mappings (Figure 10, Figure 11 and Figure 12) revealed the presence and homogeneous allocation of impregnated metals throughout the surface of TiO_2_.

**Figure 13:**
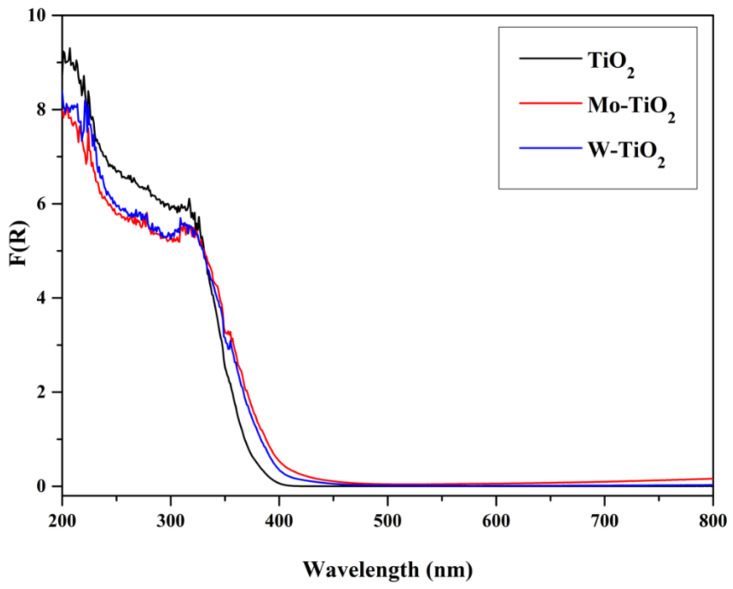
DR spectra of TiO_2_, Mo-TiO_2_, and W-TiO_2_.

**Figure 14:**
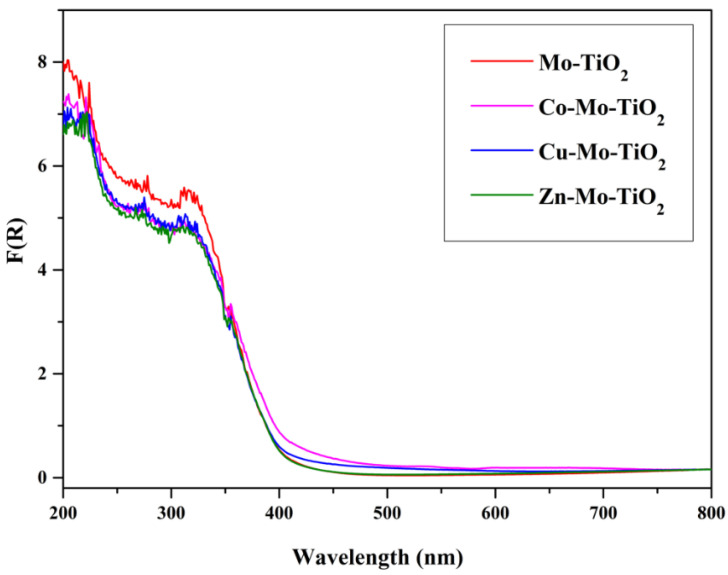
DR spectra of Mo-TiO_2_, Co-Mo-TiO_2_, Cu-Mo-TiO_2_, and Zn-Mo-TiO_2_.

**Figure 15:**
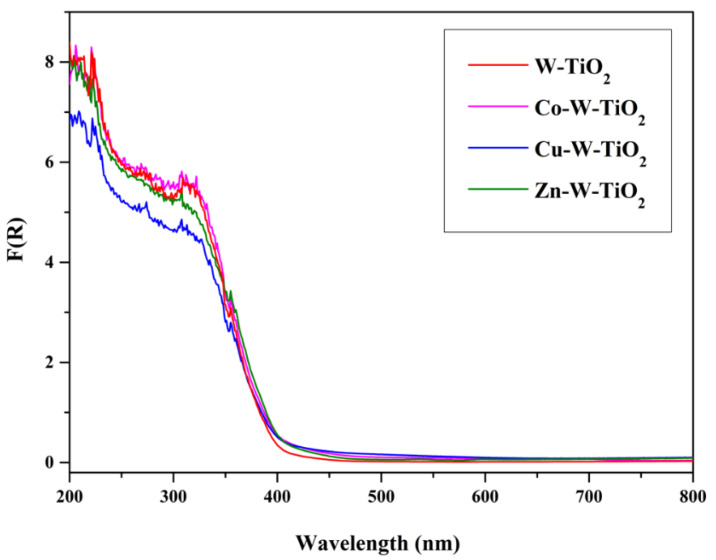
DR spectra of W-TiO_2_, Co-W-TiO_2_, Cu-W-TiO_2_, and Zn-W-TiO_2_.

The optical absorption properties of the prepared catalysts were revealed by DRS, as presented in Figure 13, Figure 14 and Figure 15. Obviously, pure TiO_2_ absorbed below 350 nm, while the incorporation of transition metals (Mo and W) into TiO_2_ induced the enhancement of the absorption capacity of near-UV light (350–450 nm). Compared with the energy gap of about 3.09 eV of undoped TiO_2_, the energy gap decreased to 2.92 eV and 2.87 eV after doping with Mo- and W-. A possible reason is the interaction between Mo or W with TiO_2_ [49,50,51]. The origin of these interactions is the formation of M1-O-Ti bonds (M1: Mo or W) and the charge transfer from Ti to Mo. These charge transfer phenomena are common in systems where an oxidic support is covered by a transition metal oxide, as in our case [40,41,52,53,54].

As can be seen, these interactions were rather higher in the case of Mo-TiO_2_, since the F(R), an analogue to absorption, was more intense for this sample, while the surface coverage seemed to be a little smaller in the case of W-TiO_2_, as the F(R) was higher in the UV region. This is in accordance with the XRD results, where crystallites of WO_3_ were detected. Both binary systems absorb less in the UV region than bare TiO_2_.

Concerning the M2-Mo-TiO_2_ samples, no significant differences could be observed (Figure 13). The coverage of TiO_2_ was higher, while, in the case of Co-Mo-TiO_2_, the adsorption in the near-UV region was higher, suggesting more intense interactions with the Co phase. Absorption in the visible region was small for the samples Co-Mo-TiO_2_ and Cu-Mo-TiO_2_, although the black color of the corresponding bulk oxides was due to the small quantity of the Co and Cu phases. This may suggest that the above oxides were rather well-dispersed on the surface of the catalyst.

The M2-W-TiO_2_ samples had similar behavior. Only the Cu-W-TiO_2_ sample had smaller absorption in the UV region, suggesting that the coverage of TiO_2_ was higher in this case.

**Figure 16:**
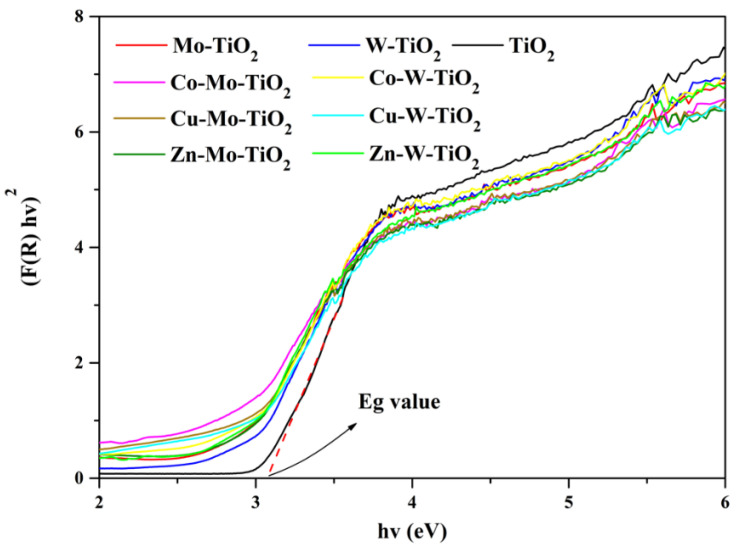
Tauc plot of all catalysts.

As was discussed, the doping of Mo-TiO_2_ and W- TiO_2_ with Co, Cu, or Zn caused the formation of a more intense peak centered at about 400 nm (Figure 14 and Figure 15) and a slight reduction in the energy gap. The energy gap (Figure 16) for Zn-Mo-TiO_2_, Cu-Mo-TiO_2_, and Co-Mo-TiO_2_ were estimated to be 2.85 eV, 2.82 eV, and 2.72 eV, respectively. Additionally, as for the Co-W-TiO_2_, Zn-W-TiO_2_, and Cu-W-TiO_2_, the Eg values were 2.87 eV, 2.86 eV, and 2.85 eV, respectively.

### 3.2. Adsorption and Photocatalytic Degradation of 4-t-BP

The adsorption and photocatalytic degradation of 4-t-BP using mono- and co-doped TiO_2_ catalysts were evaluated under dark conditions for 90 min and UV/solar light irradiation, respectively. The adsorption performance of each catalyst was identified through the determination of adsorption capacity q (mg/g) by Equation (2):

(2) $$q=\frac{\left(C_{0}-C_{e}\right)*V}{m_{\mathrm{catalyst}}}$$

where C_0_ and C_e_ represent the initial and equilibrium concentrations (mg/L) of 4-t-BP in the solution, V (L) is the volume of the 4-t-BP solution, and m _catalyst_ is the mass of the catalyst.

**Figure 17:**
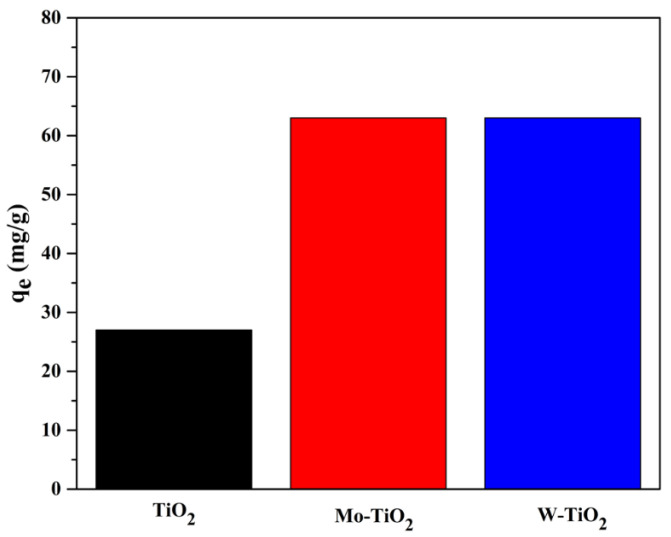
The adsorption capacities of TiO_2_, Mo-TiO_2_, and W-TiO_2_.

As shown in Figure 17, the amount of 4-t-BP adsorbed increased more than two-fold after doping TiO_2_ with Mo- or W-. At the equilibria, the adsorption capacities of Mo-TiO_2_ and W-TiO_2_ were found to be 63 mg/g for both catalysts. The enhanced adsorption capacities could be ascribed to changes in strong electrical aspects between the 4-t-BP and the doped catalyst [55]. At this point, it should be noted that the deposition of W or Mo phase increases the acidity of the surface. In a recent paper [56] about the W-TiO_2_ system, it was found that the addition of W oxo species resulted in lower point of zero values, although it was less acidic than the correspondence value for mixed oxides and changed the acid–base properties. The electron transfer between well-dispersed W phase and the TiO_2_ surface increases the surface electron density, which enhances the surface basicity of TiO_2_.

**Figure 18:**
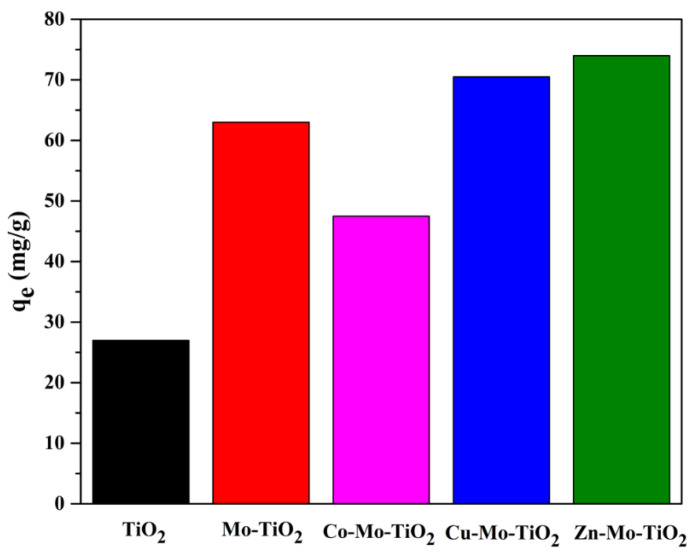
The adsorption capacities of Mo-TiO_2_, Co-Mo-TiO_2_, Cu-Mo-TiO_2_, and Zn-Mo-TiO_2_.

**Figure 19:**
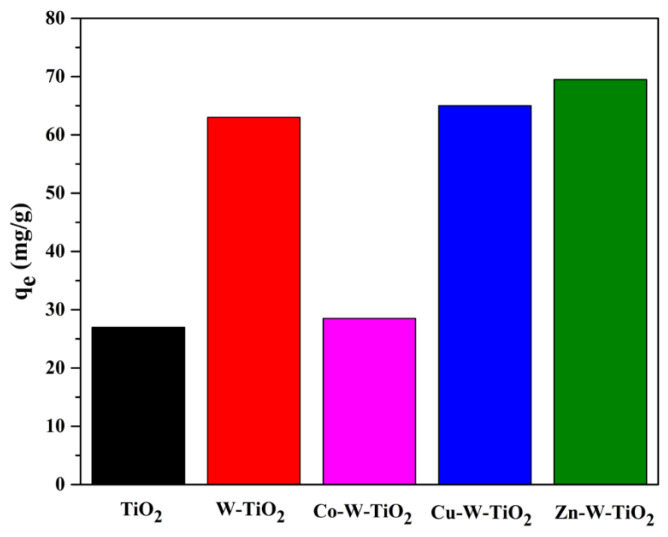
The adsorption capacities of W-TiO_2_, Co-W-TiO_2_, Cu-W-TiO_2_, and Zn-W-TiO_2_.

Further addition of Co to Mo-TiO_2_ had a negative impact on the adsorption performance, while the incorporation of Cu or Co metal ions slightly improved the adsorption of 4-t-BP (Figure 18). Similar results were obtained for doped W-TiO_2_ (Figure 19). Among all of the synthesized catalysts, Zn-doped materials exhibited the highest 4-t-BP adsorption capacity, while doping with Co had a detrimental effect on the adsorption capacity of both binary systems. Doping with Co increases the interactions between Co and Mo or W and, as a result, decreases the interactions of Mo and W oxo species with the titania surface.

It was reported [57] that surface hydroxyl groups play an important role in the surface properties of a material. These groups often have Brønsted acidity, and, therefore, they play an important role in adsorption or in photocatalytic reactions. For the Mo-TiO_2_ system, the interactions between Mo phase and TiO_2_ generate hydroxyl groups. These groups can interact with the second metal ion and immobilize it on the binary system surface.

The deposition of Co^2+^ ions on either Mo-TiO_2_ or W-TiO_2_ shifts the absorption to higher wavelengths, evidence that the Co species can be adsorbed onto the surface –OH groups, diminishing the adsorption sites for 4-t-BP. This is expected, since it is well-known that CoMo catalysts are very stable and active, especially in hydrotreatment. On the other hand, this can significantly alter the photocatalytic properties of the ternary system.

**Figure 20:**
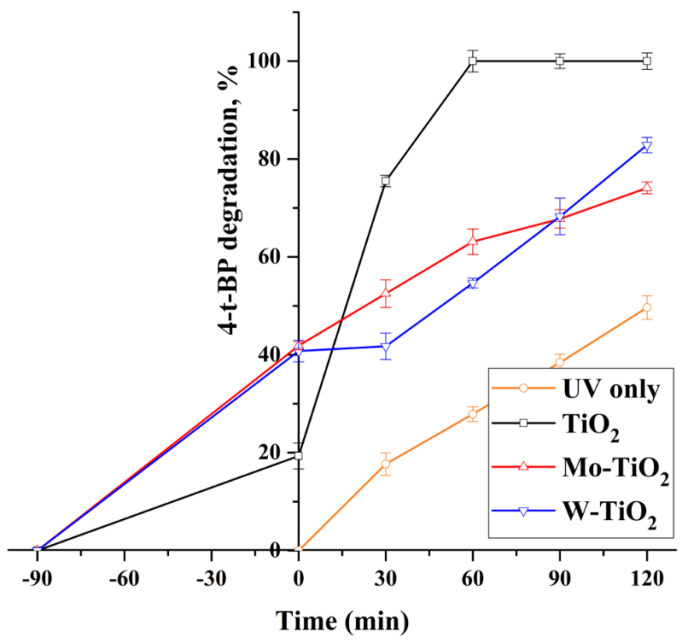
The photocatalytic activity of TiO_2_, Mo-TiO_2_, and W-TiO_2_ under UV irradiation.

In the presence of UV light, only 50% of 4-t-BP can be photodegraded in 120 min without the application of any catalyst (Figure 20). All prepared catalysts exhibited significant photodegradation when the light was on. Although the impregnation of TiO_2_ with W and Mo metals led to an improved adsorption of 4-t-BP, the photocatalytic activity of pure TiO_2_ was higher. This may be due to the higher absorption of TiO_2_ in the UV region, as was determined by DRS measurements. The results are in agreement with previous reports. For example, it was reported that the presence of various transition metals was not beneficial for the oxidation ability of the solid [58] and that only W-TiO_2_ had a positive effect on its activity. Generally, the doped catalysts exhibit recombination rates significantly higher than that of the support, which results in lower oxidation ability. Additionally, Mo deposition can have some positive effects on the activity if the deposition is not surface but subsurface [59]. In all cases, the loading of the oxoanion is crucial for the performance of the photocatalyst. Higher loadings result in lower degradation activity.

**Figure 21:**
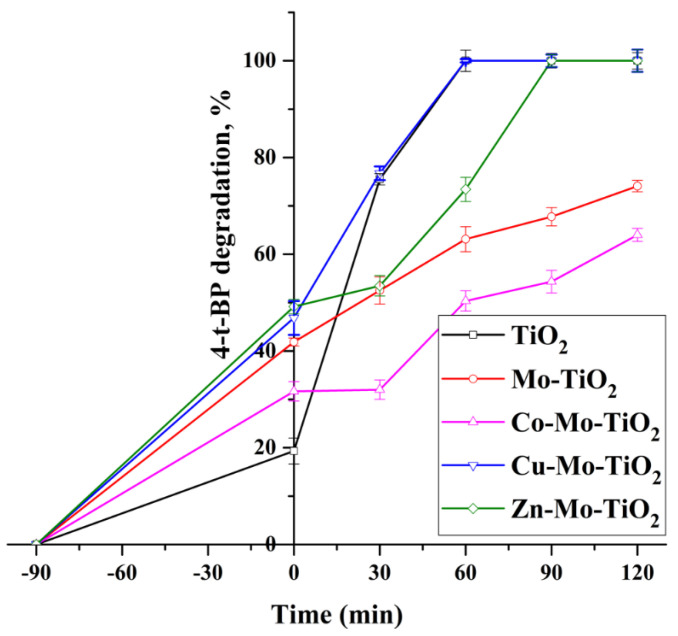
The photocatalytic activity of Mo-TiO_2_, Co-Mo-TiO_2_, Cu-Mo-TiO_2_, and Zn-Mo-TiO_2_ under UV irradiation.

**Figure 22:**
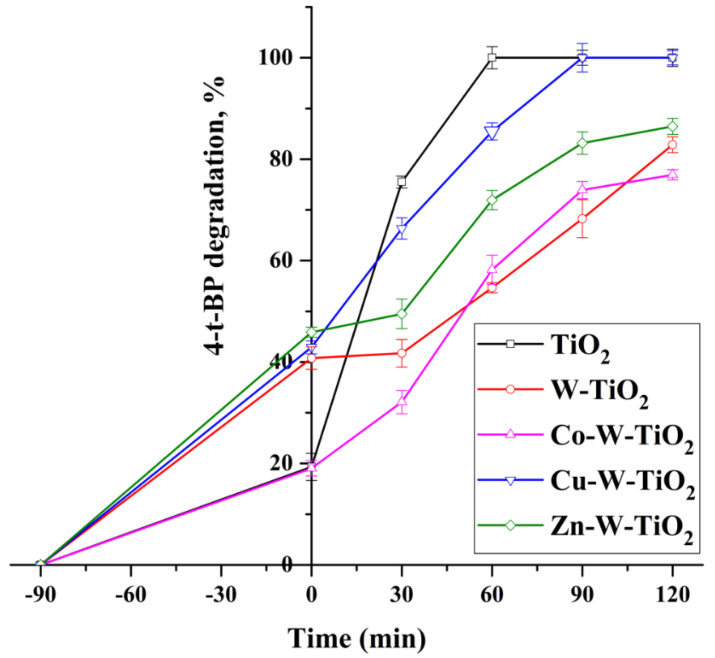
The photocatalytic activity of W-TiO_2_, Co-W-TiO_2_, Cu-W-TiO_2_, and Zn-W-TiO_2_ under UV irradiation.

The introduction of Cu, Zn, and Co metals into Mo-TiO_2_ and W-TiO_2_ resulted in different photocatalytic performances of the catalyst. The improved adsorption properties of Mo-TiO_2_ and W-TiO_2_ after doping with Cu and Zn facilitated a faster degradation of 4-t-BP (Figure 21 and Figure 22). More specifically, the incorporation of Cu into both Mo-TiO_2_ and W-TiO_2_ was favorable, where a slight 4-t-BP degradation increase was observed for Cu-Mo-TiO_2_ compared with pure TiO_2_. This is likely due to structural changes induced by the presence of Mo and Cu, which was evidenced by the high adsorption capacity and reduced energy gap coupled with the extended light absorption in the visible region.

**Figure 23:**
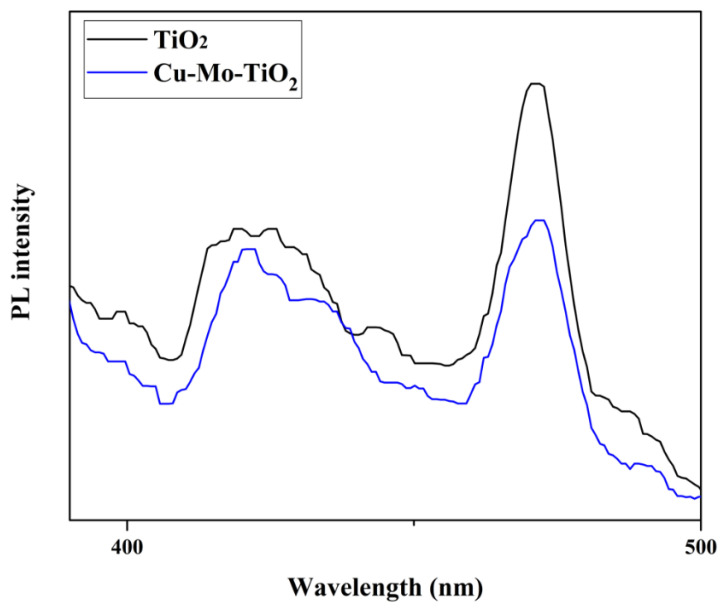
PL spectra of TiO_2_ and Cu-Mo-TiO_2_.

On the other hand, Cu-Mo-TiO_2_ showed a relatively lower PL intensity than that of pure TiO_2_ (Figure 23). This observation indicates a better charge separation, which could promote the photocatalytic performance of the catalyst.

**Figure 24:**
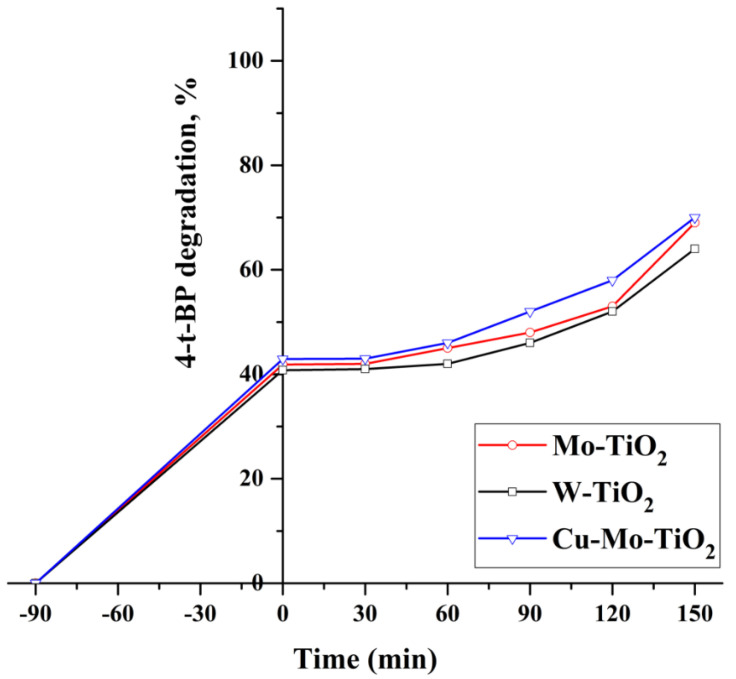
The photocatalytic activity of Cu-Mo-TiO_2_ under solar light irradiation.

Accordingly, the photocatalytic activity of Cu-Mo-TiO_2_ was investigated towards 4-t-BP degradation under solar light irradiation, and its performance was compared with that of mono-doped Mo-TiO_2_ and W-TiO_2_ catalysts (Figure 24). It was observed that the application of solar light required more time to achieve decent degradation for all tested catalysts. In 150 min of solar light exposure, about 70% of 4-t-BP could be degraded using the Cu-Mo-TiO_2_/solar system. Although the Cu-Mo-TiO_2_ catalyst exhibited better degradation efficiency, the difference was negligible in comparison to the Mo-TiO_2_ and W-TiO_2_ catalysts.

In Table 2, the results of this work are compared with previously reported ones.

## 4. Conclusions

Mono- and co-doped TiO_2_ nanoparticles with similar morphology were synthesized by simple preparation methods. The catalyst characterization evidenced that the incorporation of transition metals (Mo, W, Cu, Co, and Zn) led to homogeneous distribution of metal particles over the TiO_2_ surface and reduced the energy gap, which led to optical properties different from those of TiO_2_. Specifically, impregnation of Cu into Mo-TiO_2_ led to an increase in light absorption, particularly visible light. The catalysts were further investigated for the adsorption and photocatalytic degradation of 4-t-BP by means of UV (365 nm). Doping with transition metals increased the adsorption capacity of TiO_2_. The prepared Cu-Mo-TiO_2_ exhibited higher catalytic activity towards degradation of 4-t-BP than that of pure TiO_2_, probably due to the synergistic effect of visible light absorption, improved adsorption capacity, and suppressed electron-hole pair recombination. Complete and about 70% 4-t-BP degradation could be achieved within 60 min and 150 min using UV (365 nm) and solar light exposure, respectively.

## Figures and Tables

**Table 1:** Mean particle diameter and crystallinity of the photocatalysts.

No	Photocatalyst	Mean (Å)	% Crystallinity
1	TiO_2_	210	77.2
2	Mo-TiO_2_	199	81.2
3	Co-Mo-TiO_2_	243	82.1
4	Cu-Mo-TiO_2_	251	77.4
5	Zn-Mo-TiO_2_	240	77.7
6	W-TiO_2_	251	77.4
7	Co-W-TiO_2_	250	72.2
8	Cu-W-TiO_2_	241	73.1
9	Zn-W-TiO_2_	224	68.2

**Table 2:** Photocatalytic degradation of 4-t-BP by different materials.

Photocatalysts	Catalyst Concentration (mg/L)	4-t-BP Concentration (mg/L)	Degradation Efficiency (%)	Treatment Time (min)	Light Source and Operation Mode	Reference
Cu-Mo-TiO_2_	100	15	70	150	Solar, batch	Present work
Cu-Mo-TiO_2_	100	15	100	60	UV (365 nm), batch	Present work
Ti_2_O_3_/TiO_2_-650	200	5	89.8	150	Solar, batch	[60]
Bi_4_O_5_I_2_	1000	60	100	90	Visible, batch	[61]
Bi_12_O_17_Cl_2_/β-Bi_2_O_3_	1000	60	100	120	Visible, batch	[13]
0.5% Fe/TiO_2_	1000	30	93	60	UV (254 nm)	[15]
4% Fe/TiO_2_	200	30	87	60	UV (254 nm), continuous flow	[62]

## Data Availability

Not applicable.
